# [*N*′-(4-Meth­oxy-2-oxidobenzyl­idene)4-nitro­benzohydrazidato-κ^3^ *O*,*N*,*O*′](pyridine-κ*N*)copper(II)

**DOI:** 10.1107/S1600536808042803

**Published:** 2008-12-20

**Authors:** Nooraziah Mohd Lair, Hapipah Mohd Ali, Seik Weng Ng

**Affiliations:** a Department of Chemistry, University of Malaya, 50603 Kuala Lumpur, Malaysia

## Abstract

The pyridine-coordinated Cu^II^ atom in the title Schiff base complex, [Cu(C_15_H_11_N_3_O_5_)(C_5_H_5_N)], is *O*,*N*,*O*′-chelated by the doubly deprotonated Schiff base ligand. The metal centre is in a square-planar coordination geometry.

## Related literature

For the pyridine adducts of copper derivatives of similar ligands, see: Ali *et al.* (2004 ▶); Chen & Liu (2004 ▶); Das & Pal (2005 ▶); Fariati *et al.* (2002 ▶); Lu & Liu (2005 ▶); Lu *et al.* (2003 ▶).
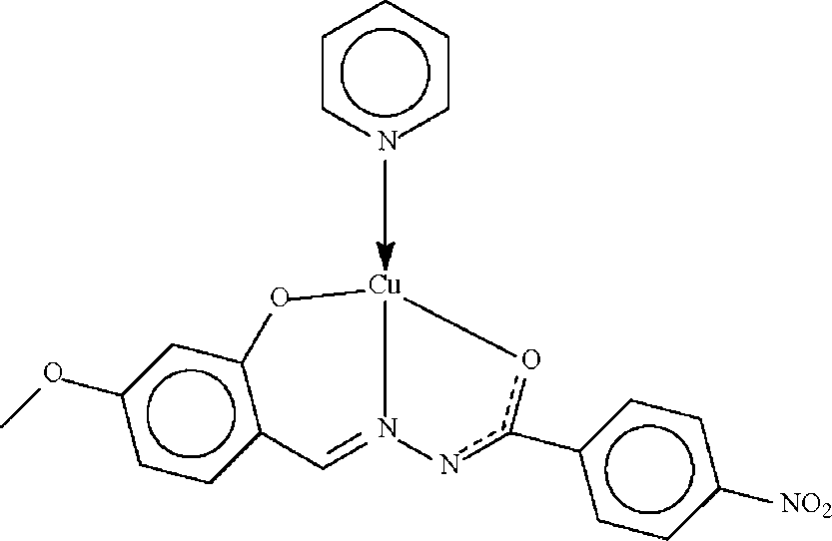


## Experimental

### 

#### Crystal data

[Cu(C_15_H_11_N_3_O_5_)(C_5_H_5_N)]
*M* *_r_* = 455.91
Triclinic, 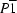

*a* = 6.3529 (1) Å
*b* = 9.8409 (2) Å
*c* = 15.1303 (3) Å
α = 98.063 (1)°
β = 92.011 (1)°
γ = 107.088 (1)°
*V* = 892.31 (3) Å^3^
*Z* = 2
Mo *K*α radiation
μ = 1.27 mm^−1^
*T* = 100 (2) K
0.40 × 0.10 × 0.05 mm

#### Data collection

Bruker SMART APEX diffractometer
Absorption correction: multi-scan (*SADABS*; Sheldrick, 1996 ▶) *T*_min_ = 0.807, *T*_max_ = 1.000 (expected range = 0.757–0.939)
6268 measured reflections
3942 independent reflections
3605 reflections with *I* > 2σ(*I*)
*R*_int_ = 0.013

#### Refinement

*R*[*F* ^2^ > 2σ(*F* ^2^)] = 0.028
*wR*(*F* ^2^) = 0.087
*S* = 1.09
3942 reflections
272 parameters
H-atom parameters constrained
Δρ_max_ = 0.45 e Å^−3^
Δρ_min_ = −0.32 e Å^−3^

### 

Data collection: *APEX2* (Bruker, 2007 ▶); cell refinement: *SAINT* (Bruker, 2007 ▶); data reduction: *SAINT*; program(s) used to solve structure: *SHELXS97* (Sheldrick, 2008 ▶); program(s) used to refine structure: *SHELXL97* (Sheldrick, 2008 ▶); molecular graphics: *X-SEED* (Barbour, 2001 ▶); software used to prepare material for publication: *publCIF* (Westrip, 2009 ▶).

## Supplementary Material

Crystal structure: contains datablocks global, I. DOI: 10.1107/S1600536808042803/bt2838sup1.cif

Structure factors: contains datablocks I. DOI: 10.1107/S1600536808042803/bt2838Isup2.hkl

Additional supplementary materials:  crystallographic information; 3D view; checkCIF report

## Figures and Tables

**Fig. 1.**
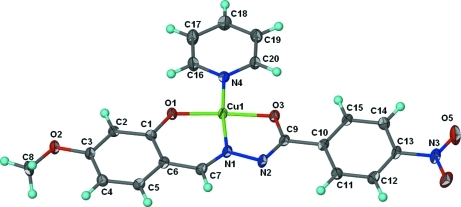
Displacement ellipsoid plot (Barbour, 2001) of Cu(C5H5N)(C15H11N3O5) at the 70% probability level. Hydrogen atoms are drawn as spheres of arbitrary radius.

## supplementary crystallographic information

### Experimental

N'-2-Hydroxy-3-methoxybenzylidene)-nitrobenzohydrazide (0.30 g, 1 mmol) and copper acetate (0.20 g, 1 mmol) were heated in a ethanol (50 ml) for 2 hours. The solvent was removed and the resulting compound recrystallized from pyridine.

### Refinement

Hydrogen atoms were placed at calculated positions (C_aromatic_–H 0.95 Å, C_methyl_–H 0.98 Å) and were treated as riding on their parent carbon atoms, with *U*(H) set to 1.2*U*eq(*C*_aromatic_) or 1.5*U*eq(*C*_methyl_).

### Crystal data

**Table:**

[Cu(C_15_H_11_N_3_O_5_)(C_5_H_5_N)]	*Z* = 2
*M**_r_* = 455.91	*F*(000) = 466
Triclinic, *P*1	*D*_x_ = 1.697 Mg m^−^^3^
Hall symbol: -P 1	Mo *K*α radiation, λ = 0.71073 Å
*a* = 6.3529 (1) Å	Cell parameters from 3716 reflections
*b* = 9.8409 (2) Å	θ = 2.4–29.2°
*c* = 15.1303 (3) Å	µ = 1.27 mm^−^^1^
α = 98.063 (1)°	*T* = 100 K
β = 92.011 (1)°	Block, brown
γ = 107.088 (1)°	0.40 × 0.10 × 0.05 mm
*V* = 892.31 (3) Å^3^	

### Data collection

**Table:**

Bruker SMART APEX diffractometer	3942 independent reflections
Radiation source: fine-focus sealed tube	3605 reflections with *I* > 2σ(*I*)
graphite	*R*_int_ = 0.013
ω scans	θ_max_ = 27.5°, θ_min_ = 1.4°
Absorption correction: multi-scan (*SADABS*; Sheldrick, 1996)	*h* = −6→8
*T*_min_ = 0.807, *T*_max_ = 1.000	*k* = −12→12
6268 measured reflections	*l* = −19→19

### Refinement

**Table:**

Refinement on *F*^2^	Primary atom site location: structure-invariant direct methods
Least-squares matrix: full	Secondary atom site location: difference Fourier map
*R*[*F*^2^ > 2σ(*F*^2^)] = 0.028	Hydrogen site location: inferred from neighbouring sites
*wR*(*F*^2^) = 0.087	H-atom parameters constrained
*S* = 1.09	*w* = 1/[σ^2^(*F*_o_^2^) + (0.0494*P*)^2^ + 0.535*P*] where *P* = (*F*_o_^2^ + 2*F*_c_^2^)/3
3942 reflections	(Δ/σ)_max_ = 0.001
272 parameters	Δρ_max_ = 0.45 e Å^−^^3^
0 restraints	Δρ_min_ = −0.31 e Å^−^^3^

### Fractional atomic coordinates and isotropic or equivalent isotropic displacement parameters (Å^2^)

**Table:**

	*x*	*y*	*z*	*U*_iso_*/*U*_eq_	
Cu1	1.14506 (4)	0.70062 (2)	0.728016 (14)	0.01312 (9)	
N1	0.9381 (3)	0.64556 (17)	0.62386 (11)	0.0150 (3)	
N2	1.0273 (3)	0.68298 (18)	0.54424 (10)	0.0159 (3)	
N3	1.7555 (3)	0.93172 (18)	0.27544 (11)	0.0189 (4)	
N4	1.3792 (3)	0.78081 (17)	0.82931 (11)	0.0145 (3)	
O1	0.9305 (2)	0.61658 (15)	0.80405 (9)	0.0163 (3)	
O2	0.2533 (2)	0.39329 (16)	0.90700 (9)	0.0199 (3)	
O3	1.3444 (2)	0.77637 (15)	0.64095 (9)	0.0159 (3)	
O4	1.6632 (3)	0.91481 (17)	0.20033 (9)	0.0247 (3)	
O5	1.9560 (3)	0.9856 (2)	0.29243 (11)	0.0311 (4)	
C1	0.7183 (3)	0.5521 (2)	0.78294 (13)	0.0139 (4)	
C2	0.5869 (3)	0.5009 (2)	0.85092 (13)	0.0152 (4)	
H2	0.6540	0.5120	0.9097	0.018*	
C3	0.3615 (3)	0.4346 (2)	0.83442 (13)	0.0149 (4)	
C4	0.2562 (3)	0.4147 (2)	0.74829 (13)	0.0151 (4)	
H4	0.1016	0.3693	0.7371	0.018*	
C5	0.3837 (3)	0.4630 (2)	0.68048 (13)	0.0146 (4)	
H5	0.3142	0.4495	0.6219	0.018*	
C6	0.6124 (3)	0.5313 (2)	0.69468 (12)	0.0140 (4)	
C7	0.7290 (3)	0.5785 (2)	0.61964 (12)	0.0149 (4)	
H7	0.6471	0.5592	0.5631	0.018*	
C8	0.0219 (3)	0.3204 (3)	0.89270 (15)	0.0247 (5)	
H8A	−0.0352	0.2939	0.9492	0.037*	
H8B	−0.0038	0.2333	0.8484	0.037*	
H8C	−0.0540	0.3840	0.8707	0.037*	
C9	1.2393 (3)	0.7493 (2)	0.56222 (12)	0.0140 (4)	
C10	1.3714 (3)	0.79538 (19)	0.48671 (12)	0.0142 (4)	
C11	1.2684 (3)	0.7902 (2)	0.40223 (13)	0.0162 (4)	
H11	1.1119	0.7555	0.3927	0.019*	
C12	1.3932 (3)	0.8352 (2)	0.33284 (13)	0.0174 (4)	
H12	1.3242	0.8321	0.2755	0.021*	
C13	1.6204 (3)	0.8849 (2)	0.34864 (12)	0.0149 (4)	
C14	1.7277 (3)	0.8905 (2)	0.43096 (13)	0.0162 (4)	
H14	1.8844	0.9243	0.4396	0.019*	
C15	1.6016 (3)	0.8457 (2)	0.50049 (12)	0.0146 (4)	
H15	1.6720	0.8492	0.5576	0.017*	
C16	1.3332 (3)	0.7615 (2)	0.91374 (13)	0.0189 (4)	
H16	1.1851	0.7146	0.9246	0.023*	
C17	1.4910 (3)	0.8068 (2)	0.98487 (13)	0.0219 (4)	
H17	1.4524	0.7913	1.0435	0.026*	
C18	1.7074 (3)	0.8753 (2)	0.96950 (14)	0.0210 (4)	
H18	1.8197	0.9079	1.0175	0.025*	
C19	1.7569 (3)	0.8955 (2)	0.88305 (13)	0.0189 (4)	
H19	1.9041	0.9416	0.8707	0.023*	
C20	1.5893 (3)	0.8476 (2)	0.81504 (13)	0.0161 (4)	
H20	1.6239	0.8625	0.7559	0.019*	

### Atomic displacement parameters (Å^2^)

**Table:**

	*U*^11^	*U*^22^	*U*^33^	*U*^12^	*U*^13^	*U*^23^
Cu1	0.01168 (13)	0.01707 (13)	0.00996 (13)	0.00263 (9)	0.00095 (8)	0.00366 (8)
N1	0.0165 (8)	0.0172 (8)	0.0117 (7)	0.0044 (6)	0.0032 (6)	0.0046 (6)
N2	0.0166 (8)	0.0193 (8)	0.0109 (7)	0.0027 (7)	0.0034 (6)	0.0048 (6)
N3	0.0245 (9)	0.0184 (8)	0.0147 (8)	0.0060 (7)	0.0075 (7)	0.0047 (6)
N4	0.0133 (8)	0.0162 (8)	0.0137 (8)	0.0039 (6)	0.0007 (6)	0.0032 (6)
O1	0.0109 (6)	0.0234 (7)	0.0136 (6)	0.0025 (5)	0.0012 (5)	0.0057 (5)
O2	0.0136 (7)	0.0297 (8)	0.0154 (7)	0.0027 (6)	0.0032 (5)	0.0082 (6)
O3	0.0139 (7)	0.0218 (7)	0.0110 (6)	0.0028 (5)	0.0006 (5)	0.0046 (5)
O4	0.0325 (9)	0.0307 (8)	0.0127 (7)	0.0103 (7)	0.0053 (6)	0.0074 (6)
O5	0.0224 (8)	0.0431 (10)	0.0229 (8)	−0.0003 (7)	0.0092 (7)	0.0092 (7)
C1	0.0131 (9)	0.0140 (8)	0.0154 (9)	0.0050 (7)	0.0014 (7)	0.0030 (7)
C2	0.0143 (9)	0.0192 (9)	0.0130 (9)	0.0056 (8)	0.0009 (7)	0.0041 (7)
C3	0.0156 (9)	0.0160 (9)	0.0141 (9)	0.0048 (7)	0.0042 (7)	0.0046 (7)
C4	0.0125 (9)	0.0156 (9)	0.0157 (9)	0.0025 (7)	0.0007 (7)	0.0018 (7)
C5	0.0167 (9)	0.0141 (8)	0.0124 (8)	0.0041 (7)	−0.0001 (7)	0.0011 (7)
C6	0.0156 (9)	0.0133 (8)	0.0127 (8)	0.0040 (7)	0.0021 (7)	0.0021 (7)
C7	0.0164 (9)	0.0153 (9)	0.0115 (8)	0.0027 (7)	−0.0009 (7)	0.0024 (7)
C8	0.0126 (10)	0.0395 (13)	0.0216 (10)	0.0032 (9)	0.0058 (8)	0.0122 (9)
C9	0.0169 (9)	0.0146 (9)	0.0116 (8)	0.0055 (7)	0.0025 (7)	0.0038 (7)
C10	0.0171 (9)	0.0129 (8)	0.0130 (9)	0.0046 (7)	0.0022 (7)	0.0034 (7)
C11	0.0146 (9)	0.0202 (9)	0.0135 (9)	0.0045 (7)	0.0005 (7)	0.0033 (7)
C12	0.0211 (10)	0.0199 (9)	0.0111 (9)	0.0057 (8)	0.0007 (7)	0.0033 (7)
C13	0.0197 (10)	0.0138 (9)	0.0116 (8)	0.0048 (7)	0.0055 (7)	0.0035 (7)
C14	0.0154 (9)	0.0159 (9)	0.0166 (9)	0.0035 (7)	0.0019 (7)	0.0021 (7)
C15	0.0168 (9)	0.0163 (9)	0.0111 (8)	0.0050 (7)	0.0014 (7)	0.0037 (7)
C16	0.0148 (10)	0.0249 (10)	0.0147 (9)	0.0021 (8)	0.0019 (7)	0.0031 (8)
C17	0.0193 (10)	0.0293 (11)	0.0125 (9)	0.0008 (9)	0.0010 (8)	0.0029 (8)
C18	0.0186 (10)	0.0248 (10)	0.0149 (9)	0.0007 (8)	−0.0034 (8)	0.0013 (8)
C19	0.0143 (9)	0.0197 (10)	0.0199 (10)	0.0008 (8)	0.0009 (8)	0.0033 (8)
C20	0.0156 (9)	0.0176 (9)	0.0143 (9)	0.0033 (7)	0.0026 (7)	0.0036 (7)

### Geometric parameters (Å, °)

**Table:**

Cu1—O1	1.8922 (14)	C6—C7	1.435 (3)
Cu1—N1	1.9239 (16)	C7—H7	0.9500
Cu1—O3	1.9320 (14)	C8—H8A	0.9800
Cu1—N4	1.9989 (16)	C8—H8B	0.9800
N1—C7	1.293 (3)	C8—H8C	0.9800
N1—N2	1.399 (2)	C9—C10	1.485 (3)
N2—C9	1.312 (3)	C10—C15	1.397 (3)
N3—O5	1.229 (2)	C10—C11	1.403 (3)
N3—O4	1.227 (2)	C11—C12	1.382 (3)
N3—C13	1.467 (2)	C11—H11	0.9500
N4—C20	1.343 (3)	C12—C13	1.381 (3)
N4—C16	1.347 (2)	C12—H12	0.9500
O1—C1	1.316 (2)	C13—C14	1.385 (3)
O2—C3	1.363 (2)	C14—C15	1.387 (3)
O2—C8	1.427 (2)	C14—H14	0.9500
O3—C9	1.299 (2)	C15—H15	0.9500
C1—C2	1.403 (3)	C16—C17	1.375 (3)
C1—C6	1.434 (3)	C16—H16	0.9500
C2—C3	1.385 (3)	C17—C18	1.386 (3)
C2—H2	0.9500	C17—H17	0.9500
C3—C4	1.405 (3)	C18—C19	1.384 (3)
C4—C5	1.380 (3)	C18—H18	0.9500
C4—H4	0.9500	C19—C20	1.381 (3)
C5—C6	1.404 (3)	C19—H19	0.9500
C5—H5	0.9500	C20—H20	0.9500
			
O1—Cu1—N1	93.57 (6)	O2—C8—H8B	109.5
O1—Cu1—O3	174.58 (6)	H8A—C8—H8B	109.5
N1—Cu1—O3	81.17 (6)	O2—C8—H8C	109.5
O1—Cu1—N4	92.67 (6)	H8A—C8—H8C	109.5
N1—Cu1—N4	172.51 (7)	H8B—C8—H8C	109.5
O3—Cu1—N4	92.68 (6)	O3—C9—N2	125.19 (17)
C7—N1—N2	117.25 (16)	O3—C9—C10	117.01 (17)
C7—N1—Cu1	127.41 (13)	N2—C9—C10	117.79 (16)
N2—N1—Cu1	115.34 (12)	C15—C10—C11	119.71 (17)
C9—N2—N1	108.05 (15)	C15—C10—C9	119.31 (16)
O5—N3—O4	123.25 (17)	C11—C10—C9	120.98 (18)
O5—N3—C13	118.27 (16)	C12—C11—C10	120.41 (18)
O4—N3—C13	118.47 (17)	C12—C11—H11	119.8
C20—N4—C16	117.86 (17)	C10—C11—H11	119.8
C20—N4—Cu1	121.38 (13)	C11—C12—C13	118.53 (17)
C16—N4—Cu1	120.64 (14)	C11—C12—H12	120.7
C1—O1—Cu1	127.54 (12)	C13—C12—H12	120.7
C3—O2—C8	117.33 (15)	C12—C13—C14	122.62 (18)
C9—O3—Cu1	110.23 (12)	C12—C13—N3	119.26 (17)
O1—C1—C2	118.11 (17)	C14—C13—N3	118.12 (18)
O1—C1—C6	124.11 (17)	C15—C14—C13	118.62 (18)
C2—C1—C6	117.78 (17)	C15—C14—H14	120.7
C3—C2—C1	121.55 (17)	C13—C14—H14	120.7
C3—C2—H2	119.2	C14—C15—C10	120.11 (17)
C1—C2—H2	119.2	C14—C15—H15	119.9
O2—C3—C2	115.33 (17)	C10—C15—H15	119.9
O2—C3—C4	123.65 (18)	N4—C16—C17	122.91 (19)
C2—C3—C4	121.01 (18)	N4—C16—H16	118.5
C5—C4—C3	118.12 (18)	C17—C16—H16	118.5
C5—C4—H4	120.9	C16—C17—C18	118.84 (19)
C3—C4—H4	120.9	C16—C17—H17	120.6
C4—C5—C6	122.53 (17)	C18—C17—H17	120.6
C4—C5—H5	118.7	C19—C18—C17	118.81 (19)
C6—C5—H5	118.7	C19—C18—H18	120.6
C5—C6—C1	119.00 (17)	C17—C18—H18	120.6
C5—C6—C7	117.98 (17)	C20—C19—C18	119.05 (19)
C1—C6—C7	123.02 (17)	C20—C19—H19	120.5
N1—C7—C6	124.30 (17)	C18—C19—H19	120.5
N1—C7—H7	117.9	N4—C20—C19	122.52 (18)
C6—C7—H7	117.9	N4—C20—H20	118.7
O2—C8—H8A	109.5	C19—C20—H20	118.7
			
O1—Cu1—N1—C7	1.77 (18)	C5—C6—C7—N1	178.35 (18)
O3—Cu1—N1—C7	−179.56 (18)	C1—C6—C7—N1	−1.0 (3)
O1—Cu1—N1—N2	−178.07 (13)	Cu1—O3—C9—N2	1.5 (2)
O3—Cu1—N1—N2	0.60 (12)	Cu1—O3—C9—C10	−177.59 (12)
C7—N1—N2—C9	−179.91 (17)	N1—N2—C9—O3	−1.0 (3)
Cu1—N1—N2—C9	−0.05 (19)	N1—N2—C9—C10	178.08 (15)
O1—Cu1—N4—C20	174.53 (15)	O3—C9—C10—C15	9.0 (3)
O3—Cu1—N4—C20	−4.54 (15)	N2—C9—C10—C15	−170.09 (17)
O1—Cu1—N4—C16	−1.39 (16)	O3—C9—C10—C11	−170.48 (17)
O3—Cu1—N4—C16	179.54 (15)	N2—C9—C10—C11	10.4 (3)
N1—Cu1—O1—C1	−2.34 (16)	C15—C10—C11—C12	−0.4 (3)
N4—Cu1—O1—C1	173.51 (16)	C9—C10—C11—C12	179.15 (17)
N1—Cu1—O3—C9	−1.01 (12)	C10—C11—C12—C13	0.1 (3)
N4—Cu1—O3—C9	−176.72 (13)	C11—C12—C13—C14	0.4 (3)
Cu1—O1—C1—C2	−178.09 (13)	C11—C12—C13—N3	179.37 (17)
Cu1—O1—C1—C6	1.7 (3)	O5—N3—C13—C12	174.64 (18)
O1—C1—C2—C3	178.63 (17)	O4—N3—C13—C12	−5.6 (3)
C6—C1—C2—C3	−1.1 (3)	O5—N3—C13—C14	−6.3 (3)
C8—O2—C3—C2	−178.07 (18)	O4—N3—C13—C14	173.44 (18)
C8—O2—C3—C4	2.8 (3)	C12—C13—C14—C15	−0.6 (3)
C1—C2—C3—O2	−178.47 (17)	N3—C13—C14—C15	−179.62 (16)
C1—C2—C3—C4	0.7 (3)	C13—C14—C15—C10	0.4 (3)
O2—C3—C4—C5	179.17 (17)	C11—C10—C15—C14	0.1 (3)
C2—C3—C4—C5	0.0 (3)	C9—C10—C15—C14	−179.40 (17)
C3—C4—C5—C6	−0.4 (3)	C20—N4—C16—C17	−0.1 (3)
C4—C5—C6—C1	−0.1 (3)	Cu1—N4—C16—C17	175.93 (16)
C4—C5—C6—C7	−179.44 (17)	N4—C16—C17—C18	0.0 (3)
O1—C1—C6—C5	−178.95 (17)	C16—C17—C18—C19	−0.1 (3)
C2—C1—C6—C5	0.8 (3)	C17—C18—C19—C20	0.4 (3)
O1—C1—C6—C7	0.4 (3)	C16—N4—C20—C19	0.4 (3)
C2—C1—C6—C7	−179.86 (17)	Cu1—N4—C20—C19	−175.60 (15)
N2—N1—C7—C6	179.34 (16)	C18—C19—C20—N4	−0.6 (3)
Cu1—N1—C7—C6	−0.5 (3)		
